# Preharvest Application of Chitosan Improves the Postharvest Life of ‘Garmrok’ Kiwifruit through the Modulation of Genes Related to Ethylene Biosynthesis, Cell Wall Modification and Lignin Metabolism

**DOI:** 10.3390/foods10020373

**Published:** 2021-02-09

**Authors:** H. M. Prathibhani C. Kumarihami, Jin Gook Kim, Yun-Hee Kim, Mockhee Lee, Young-Suk Lee, Yong-Bum Kwack, Joonyup Kim

**Affiliations:** 1 Division of Applied Life Science, Graduate School, Gyeongsang National University, Jinju 52828, Korea; prathibhanick@gmail.com (H.M.P.C.K.); jgkim119@gnu.ac.kr (J.G.K.); 2 Institute of Agriculture and Life Science, Gyeongsang National University, Jinju 52828, Korea; 3 Department of Biology Education, Gyeongsang National University, Jinju 52828, Korea; cefle@gnu.ac.kr; 4 Namhae Branch, National Institute of Horticultural and Herbal Science, Rural Development Administration, Namhae 52430, Korea; mockey92@korea.kr; 5 Department of Horticulture Research, Gyeongsangnam-do Agricultural Research and Extension Service, Jinju 52733, Korea; yseve77@korea.kr; 6 Department of Fruit Science, Korea National College of Agriculture and Fisheries, Jeonju 54874, Korea; kwack@korea.kr; 7 Department of Horticultural Science, Chungnam National University, Daejeon 34134, Korea

**Keywords:** cell wall modification, chitosan, ethylene biosynthesis, fruit quality, lignin metabolism, postharvest quality, preharvest treatment

## Abstract

The influence of the preharvest application of chitosan on physicochemical properties and changes in gene expression of ‘Garmrok’ kiwifruit during postharvest cold storage (0 °C; RH 90–95%; 90 days) was investigated. Preharvest treatment of chitosan increased the fruit weight but had no significant effect on fruit size. The chitosan treatment suppressed the ethylene production and respiration rate of kiwifruit during the cold storage. The reduction of ethylene production of chitosan-treated kiwifruit was accompanied with the suppressed expression of ethylene biosynthesis genes. Moreover, preharvest application of chitosan diminished weight loss and delayed the changes in physicochemical properties that include firmness, soluble solids content, titratable acidity, total sugars, total acids, total phenols, and total lignin. As a result, the preharvest application of chitosan delayed the maturation and ripening of fruit. Expression of genes related to cell wall modification was down-regulated during the early maturation (ripening) period, while those related to gene expression for lignin metabolism were up-regulated at the later stages of ripening. These results demonstrate that the preharvest application of chitosan maintained the fruit quality and extends the postharvest life of ‘Garmrok’ kiwifruit, possibly through the modulation of genes related to ethylene biosynthesis, cell wall modification, and lignin metabolism.

## 1. Introduction

Kiwifruit (*Actinidia* sp.) is widely accepted by consumers for its organoleptic and nutritional properties. It is an excellent source of vitamin C (L-ascorbic acid) [1,2], and is generally known as “Chinese gooseberry”, “China’s miracle fruit”, and “the horticultural wonder of New Zealand” [3]. The genus *Actinidia* contains more than 60 species with a wide diversity, but only two of them (*A. deliciosa*, and *A. chinensis*) are being produced commercially [2,4,5]. Due to their high nutritional value and desirable taste, the demand for kiwifruits has been increasing. Year-round production and supply of kiwifruit to meet the demands inevitably requires proper postharvest management.

Kiwifruit is extremely perishable, with a typical climacteric ripening pattern [1,5]. It is harvested at an unripe but physiologically mature stage [5]. After harvest, the physicochemical properties of fruit decline rapidly due to the influence of internal biochemical reactions and the external environment [6,7]. The postharvest performance of kiwifruit is strongly affected by the maturity or physiological state of the fruit at harvest, in conjunction with the applied postharvest management. In particular, the postharvest storage life of kiwifruit is mostly limited by its relatively high metabolic activity and extreme sensitivity to ethylene during storage [5,6,7,8]. Accordingly, the conditions for the preservation of kiwifruit for prolonged periods are undoubtedly important. The kiwifruit industry highly relies on low temperature [5,9] in combination with controlled/modified atmospheres [7,10] to extend their postharvest life. Besides, several other preservation technologies including edible coatings [7,10,11], and treatments with chemical agents such as methyl jasmonate [12], salicylic acid [6], and 1-methyl cyclopropane (1-MCP) [12] have been trying to extend the kiwifruit postharvest life. Among these, the application of edible coatings has been reported as one of the novel technologies with great potential for extending the postharvest life of kiwifruit.

The edible coatings on fresh fruit serve as an alternative to modified atmosphere packaging, as they reduce the quality changes and quantity losses through control of the internal atmosphere of the individual fruit [13]. It has been shown that edible coatings have the potential to reduce moisture loss, rate of respiration, ethylene production, and ripening, while they maintain quality along with storability [7,11,13,14]. In postharvest management, polysaccharide-, protein-, and lipid-based solutions have been demonstrated as applicable edible coatings that prolonged the postharvest life of the whole kiwifruit or other fruits [1,7,10,11,14,15,16].

Recently, the natural compound chitosan (poly *β*-(1,4) *N*-acetyl-d-glucosamine) has been widely used as an edible coating. It is a polysaccharide derived from the deacetylation of chitin [6,17,18,19,20]. It has been shown that the use of chitosan as an edible coating enhances the quality and postharvest life of various fruits owing to its excellent film-forming, non-toxic, biocompatible, biodegradable, and antifungal properties [2,8,18,20,21,22]. Besides, several studies have demonstrated that the postharvest use of chitosan as an edible coating maintains quality and extends the postharvest life of kiwifruits such as green-fleshed (*A. deliciosa*) kiwifruit [3,18,23], yellow-fleshed kiwifruit (*A. chinensis*) [6,24], red kiwifruit (*A. melanandra*) [2], hardy kiwifruit (*A. arguta*) [13], and arctic kiwifruit (*A. kolomikta*) [21]. Although chitosan can be applied in either preharvest or postharvest treatments, the reports on preharvest treatments of chitosan and the effects on the postharvest control are still limited. Nonetheless, the preharvest application of chitosan is highly feasible and can be applied on the fruit around harvest time [19].

In our previous studies, we evaluated the effectiveness of the application of preharvest chitosan in combination with calcium chloride (Ca-chitosan) that resulted in the enhancement of fruit quality and postharvest life of hardy kiwifruit (*A. arguta* ‘Saehan’) [25] and green-fleshed kiwifruit (*A. deliciosa* ‘Garmrok’) [8]. In a separate study, Zhang et al. [22] reported that the preharvest spraying of chitosan composite films (chitosan, calcium, dextrin, ferulic acid, and auxiliaries) had several positive effects on the postharvest quality and control of diseases in kiwifruit (*A. deliciosa* ‘Guichang’). Although these results suggest the efficacy of preharvest treatment of chitosan in maintaining the postharvest properties of kiwifruit, the physiological changes and underlying molecular events involved in maintaining fruit physicochemical properties need to be further investigated. In the current study, we show that the preharvest application of chitosan maintains the fruit quality and extends the postharvest life of ‘Garmrok’ kiwifruit possibly through the modulation of genes related to ethylene biosynthesis, cell wall modification, and lignin metabolism.

## 2. Materials and Methods

### 2.1. Plant Materials and Preharvest Chitosan Treatment

The study was carried out from 4 October 2019 to 3 February 2020, on kiwifruit (*A. deliciosa* ‘Garmrok’). Kiwifruit was harvested from an orchard in Namhae Sub-Station, National Institute of Horticultural and Herbal Science, Rural Development Administration, Korea. ‘Garmrok’ kiwifruit vines were cultivated on a pergola trellis system and general kiwifruit cultivation recommendations were implemented.

The experiment comprised three treatments that included control (0), 100, and 500 mg·L^−1^ chitosan. Untreated fruits served as the control treatment. Chitosan (from shrimp shells; ≥75% deacetylation; Sigma-Aldrich, Seoul, Korea) at a concentration of 20 g·L^−1^ was dissolved in distilled water consisting of 0.1 mol·L^−1^ acetic acids by stirring to make a stock solution. The resultant stock solution was further diluted with distilled water to make working solutions at 100 and 500 mg·L^−1^ concentrations. Tween 80 (0.1%) was added to improve wettability. The chitosan concentrations used in the study were chosen based on our previous studies [8,25]. Each treatment comprised three biological replicates (trees) in a randomized complete block design in which a single tree corresponds to the experimental unit. Kiwifruit on vines was fully dipped in a cup of chitosan solution until the fruit were completely wet. The chitosan was applied at four times: 4 October 2019 (146 days after full bloom (DAFB)), 12 October 2019 (154 DAFB), 19 October 2019 (161 DAFB), and 28 October 2019 (170 DAFB). The fruit from each treatment were harvested on 2 November 2019 (175 DAFB).

Kiwifruit were culled out for uniformity of size and lacking defects. The selected fruit were packed in corrugated cardboard boxes (10 kg capacity) laid with a perforated polyethylene film liner. All experiments were performed in triplicate. Fruit were stored for 90 days at 0 °C with relative humidity (RH) of 90–95%. The fruit physicochemical attributes and relative gene expressions were evaluated at 30-day intervals.

### 2.2. Physicochemical Quality Attributes of Fruit

Kiwifruit weight, size, core firmness, and flesh firmness were measured for 10 biological replicates (fruit) according to our previous study [8]. A rheometer (RHEO TEX SD-700, Sun Scientific Inc., Tokyo, Japan) fitted with an 8 mm round, flat-ended probe, compressing at a depth of 3 mm and a crosshead speed of 120 mm·min^−1^ was used to measure fruit firmness. The fruit were sliced into longitudinal halves and each half was measured for flesh firmness in the central zone after the peel (~2 mm thick) was removed. The fruit were cut through the equator (2 cm radial slice) and the core tissue was measured for core firmness [8]. Firmness was represented in Newton (N). Fruit weight loss was measured using a digital balance from the beginning to the end of the storage and expressed as the percentage of weight loss relative to the initial weight.

Changes in respiration rate and ethylene production rate were measured in triplicate using a gas chromatograph. After weighing the individual fruit, each of them was placed in a 630 mL volume airtight polypropylene container (HPL851-2.1L, Locknlock, Seoul, Korea) fitted with a rubber septum, for 3 h at room temperature. Air samples of the headspace were removed from the septum with a syringe and injected into a gas chromatograph (GC-7890B; Agilent Technologies, Santa Clara, CA, USA) that is equipped with a stainless steel column (2.0 m × 3.0 mm i.d.) packed with Porapak Q (Shinwa, Kyoto, Japan) and a flame ionization detector (FID) to measure the ethylene production. The respiration rate was determined using a gas chromatograph (GC 6890, Agilent Technologies, USA) that is equipped with a stainless steel column (2.0 m × 3.0 mm i.d.) packed with Shincarbon ST (Shinwa, Kyoto, Japan) and a thermal conductivity detector (TCD). The measurements were expressed in mg·CO_2_ kg^−1^ h^−1^ and μL·C_2_H_4_ kg^−1^ h^−1^.

Surface of preharvest chitosan-treated and untreated ‘Garmrok’ kiwifruit peel was examined by scanning electron microscopy (SEM, LEO 1420VP). The micrographs were viewed at an accelerating voltage of 20 kV in high vacuum conditions. Before SEM observation, the samples were dried using a freeze dryer at −78 °C for 2 days (Ilshin BioBase, Gyeonggi-do, Korea). The dried samples were coated with an ultrathin layer of palladium/gold layer (Pd/Au) on an ion sputtering machine (Quorum Techn, SC7620) and comparable magnifications of kiwifruit peel of coated and non-coated fruits were photographed.

The soluble solids content (SSC, %) and titratable acidity (TA, %) were measured as described in our previous study [8]. Ten biological replicates were measured for SSC, while TA was measured in three replications in which one replicate contained the extract of ten fruits. The contents of total sugar, organic acid, phenolic, and lignin were measured in three replications in which one replicate contained the extract of three fruits. Sugars (fructose, glucose, and sucrose) and organic acids (oxalic, quinic, malic, and citric) were analyzed by HPLC (Agilent 1200 Chemstation, Agilent, CA, USA), according to the method of Kim et al. [4] with slight modifications. A 250 mm × 4.6 mm i.d. Shodex Asahipak NH2P-50 4E column (Showa Denko, Tokyo, Japan) was used for sugar analysis. The organic acid analysis was done using a 250 mm × 4.6 mm i.d. Develosil RPAQUEOUS-AR-3 column (Nomura Chemical Co., Ltd., Seto, Japan). The peaks were detected at 214 nm with UV/VIS detector (1200 Variable Wavelength Detector, Agilent, CA, USA). Sugars and organic acids were detected by the retention time in the chromatograms compared to the standards. The results were expressed in g/100 g on a fresh weight basis. The total sugar content was detected in a mixture of fructose, glucose, and sucrose (Table S1). The total organic acid was detected in a mixture of oxalic, quinic, malic, and citric acids (Table S1).

The content of total phenolic was analyzed spectrophotometrically using the modified Folin–Ciocâlteu method, following Kim et al. [26]. The absorbance was recorded at 725 nm using a UV–vis spectrophotometer (Varioskan Flash Multimode Reader, Thermo Fisher Scientific, Waltham, MA, USA). The content of total phenolic was calculated using a standard curve of gallic acid and expressed as mg of gallic acid equivalent (GAE) per 100 g on a fresh weight basis.

The total lignin content of kiwifruit flesh was assessed by a two-step acid hydrolysis procedure following the methodology of the National Renewable Energy Laboratory (NREL/TP-510-42618) [27]. The primary hydrolysis step was done with 72% H_2_SO_4_ at 30 °C for 60 min. At the secondary hydrolysis step, the reaction mixture was diluted to 4% H_2_SO_4_ and autoclaved at 121 °C for 1 h. The autoclaved hydrolysis solution was vacuum filtered into the previously weighed crucibles of medium porosity (10 to 15 µm). The solid residue remaining in the filter crucibles was oven-dried at 105 °C overnight and is considered to be the acid-insoluble lignin. The content of acid-soluble lignin in the hydrolysates was also quantified. The acid-soluble lignin was calculated based on measuring absorbance at 320 nm in a UV-visible spectrophotometer (UV–vis, 7205, Jenway Co., Staffordshire, UK). The content of total lignin in the sample was determined as the sum of the acid-insoluble lignin and acid-soluble lignin, expressed as a percentage.

### 2.3. RNA Extraction, cDNA Synthesis, and Gene Expression Analysis by Quantitative Real-Time PCR

The total RNA of kiwifruit flesh tissue was isolated from three biological replicates of fruit at every 30 days of storage using the Ribospin^TM^ Plant Total RNA Purification Kit (GeneAll Biotechnology Co., Ltd., Seoul, Korea) by following the manufacturer’s protocol. The extracted RNA was quantified by Nanodrop spectrophotometer (Thermo Scientific™ NanoDrop 2000, Waltham, MA, USA). One microgram of total RNA was used for cDNA synthesis using the TOPscript^TM^ RT DryMIX (*dT18 plus*) Kit (Enzynomics; Republic of Korea) according to the manufacturer’s protocol.

The real-time qPCR was performed with gene-specific primers (Table S2). Each reaction was performed in triplicate. For each sample in 20 µL final volume were contained 1 µL cDNA, 1 µL specific primers, and 10 µL of 2X Real-Time PCR Master Mix (Including SFCgreen^®^ I in the mixture) according to the manufacturer’s protocol (BioFACT^TM^, Yuseong-Gu, Daejeon, Korea).

Three genes of cell wall modification, *A. deliciosa* polygalacturonase C (*AdPGC*), *A. deliciosa* expansin 1 (*AdEXP1*), *A. deliciosa* expansin 2 (*AdEXP2*), and two genes of ethylene biosynthesis, *A. deliciosa* ACC (1-aminocyclopropane-1-carboxylic acid) synthase 2 (*AdACS2*), *A. deliciosa* ACC oxidase 2 (*AdACO2*) were selected based on our previous studies [28,29]. The lignin metabolism-related genes, *A. chinensis* phenylalanine ammonia-lyase (*AcPAL*), *A. chinensis* cinnamyl-alcohol dehydrogenase (*AcCAD*), and *A. chinensis* peroxidase 2 (*AcPOD2*) were selected according to Li et al. [12]. For cell wall modification genes and ethylene biosynthesis genes quantitative RT-PCR was performed using the Rotor-Gene Q detection system (Qiagen, Hilden, Germany) and included initial annealing of 5 min at 95 °C, followed by 50 cycles of 15 s at 95 °C, 30 s at 60 °C, and 45 s at 72 °C [28,29], followed by a melting-curve analysis at the end of each run. The cycling procedure for lignin metabolism-related genes included initial annealing of 3 min at 95 °C, followed by 44 cycles of 10 s at 95 °C, 30 s at 57 °C, and 30 s at 72 °C [12]. The kiwifruit actin gene, *A. deliciosa* actin 1 (*AdACT1*) was used as the internal control (housekeeping gene) to normalize expression differences in each sample. Quantitative RT-PCR data were analyzed using the 2^−ΔΔCT^ method [30], and changes in gene expression caused by chitosan treatment was compared to untreated fruit (control) of each sampling date (*p* ≤ 0.05).

### 2.4. Statistical Analysis

The data were subjected to analysis of variance (ANOVA) using statistical analysis software (SAS, version 9.4, SAS Institute Inc., Cary, NC, USA). The significance of differences between the mean values were determined by least significant difference (LSD) test (*p* ≤ 0.05). Statistical analysis of qRT-PCR data was performed with Student’s *t*-test (*p* ≤ 0.05). A two-way ANOVA compared the independent groups (preharvest treatment and storage days at 0 °C) based on the dependent variables as shown in Supplementary Tables S3–S6. The graphics of the analyzed results were generated using SigmaPlot v12.0 (Systat Software, Inc., SigmaPlot for Windows, San Jose, CA, USA).

## 3. Results

### 3.1. Fruit Weight and Size at Harvest

The chitosan-treated fruit had the highest fruit weight of 86.27 and 91.79 g (*p* ≤ 0.05) at 100 and 500 mg·L^−1^ concentrations, respectively (Table 1) when compared with the average weight of the control fruit (81.49 g). The fruit size was measured by means of fruit length, longitudinal width, and transverse width. There was no difference in fruit length and longitudinal width between the treatments, except for the transverse width (Table 1). The smaller values of transverse width of 50.28 and 51.86 mm were measured for fruit treated with 100 and 500 mg·L^−1^ chitosan, respectively, and a larger value of transverse width was observed for the control fruit (52.06 mm).

### 3.2. Ethylene Production (C_2_H_4_) and Respiration (CO_2_) Rates

**Figure 1:**
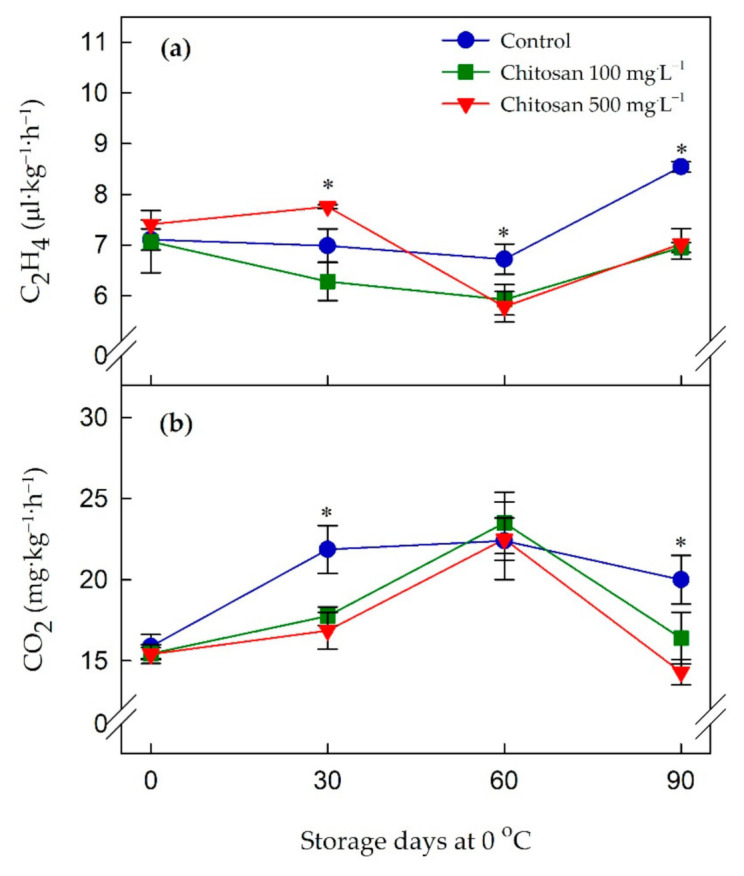
Effects of preharvest chitosan application on (**a**) ethylene production and (**b**) respiration rates during cold storage in ‘Garmrok’ kiwifruit. Vertical bars indicate SE with n = 3. * indicates significant differences between treatments at each sampling date, according to the least significant difference (LSD) test at *p* ≤ 0.05.

The differences were not found in either C_2_H_4_ production or respiration rates (CO_2_ production) at harvest date (Figure 1a,b). After 30 days of storage, C_2_H_4_ production was highest (*p* ≤ 0.05) in fruit treated with 500 mg·L^−1^ chitosan while it was lowest (*p* ≤ 0.05) in fruit treated with 100 mg·L^−1^ chitosan (Figure 1a). For the rest of the storage period, C_2_H_4_ production was lower (*p* ≤ 0.05) for both 100 and 500 mg·L^−1^ chitosan-treated fruit than that for the control samples. As for the respiration, control fruit displayed the highest respiration rate during the cold storage than the preharvest chitosan-treated fruit (Figure 1b). While there was a lower CO_2_ production with the higher chitosan concentration, the mean values of CO_2_ production in 100 and 500 mg·L^−1^ chitosan pre-treated fruit were not statistically significant. At the end of the storage, the highest CO_2_ production was found in control fruit (20.0 mg·kg^−1^·h^−1^), followed by 100 mg·L^−1^ (16.4 mg·kg^−1^·h^−1^) and 500 mg·L^−1^ (14.3 mg·kg^−1^·h^−1^) chitosan-treated kiwifruit. Moreover, a significant effect of the days of storage at 0 °C was observed for C_2_H_4_ production and respiration rates (Table S3).

### 3.3. Loss of Fruit Weight

The loss of fruit weight for all treatments displayed a similar increasing pattern as the storage period proceeded (Table 2 and Table S3). Preharvest chitosan application (100 and 500 mg·L^−1^) deterred the weight loss of ‘Garmrok’ kiwifruit during the cold storage, and the highest (*p* ≤ 0.05) weight loss was observed in the control fruit (Table 2). Although the weight loss appeared to be slightly higher at 500 mg·L^−1^ chitosan than 100 mg·L^−1^ chitosan, they were not statistically significant. The greatest loss of fruit weight for each treatment was observed at the end of storage (90 days), as shown as weight loss of 1.8%, 2.2%, and 2.3% for 100, 500 mg·L^−1^ chitosan and control treatment, respectively.

### 3.4. Changes in Soluble Solids Content, Titratable Acidity, Total Sugars, and Total Acids

As expected, soluble solids content (SSC) and total sugar content increased over the period of storage for all the treatments, which reached the maximum value at the end of storage (Table 3 and Table S4). Nonetheless, the preharvest treatment of chitosan was associated with slower increasing rates of SSC during storage, as significantly lower (*p* ≤ 0.05) SSC at the end of storage were observed in preharvest chitosan-treated fruit (11.9% in 100 mg·L^−1^ and 11.6% in 500 mg·L^−1^) compared with the control fruit (12.7%). In addition, preharvest chitosan-treated fruit displayed the lowest increase in total sugar content during the storage which resulted in a significantly lower (*p* ≤ 0.05) total sugar content at the end of storage. At harvest, the ratio of fructose + glucose/sucrose, a potential indicator of invertase activity [31], was lower (*p* ≤ 0.05) in 100 and 500 mg·L^−1^ chitosan-treated fruit. However, there was no difference in the fructose + glucose/sucrose ratio among treatments for the rest of the storage.

In regard to titratable acidity (TA) and total acid content, it was clear that both attributes displayed a decreasing trend over the storage time (Table 3 and Table S4). The preharvest chitosan-treated fruit had the greatest TA values (*p* ≤ 0.05) at harvest. The fruit treated with 100 mg·L^−1^ chitosan displayed the positive effect on TA that preserved the greatest content (*p* ≤ 0.05) during storage time compared with the control and 500 mg·L^−1^ chitosan-treated fruit. As for the total acid content, preharvest chitosan treatments resulted in higher (*p* ≤ 0.05) values at harvest time than the control. Throughout the whole storage, the application of 100 mg·L^−1^ chitosan was most effective in preserving the greatest total acid content, followed by 500 mg·L^−1^ chitosan when compared with the control. In addition, the application of preharvest chitosan with 100 and 500 mg·L^−1^ caused the greatest (*p* ≤ 0.05) citric acid/quinic acid ratio at harvest; however, there was no significant difference in this ratio among treatments during the rest of storage.

### 3.5. Changes in Total Phenolic and Lignin Contents

**Figure 2:**
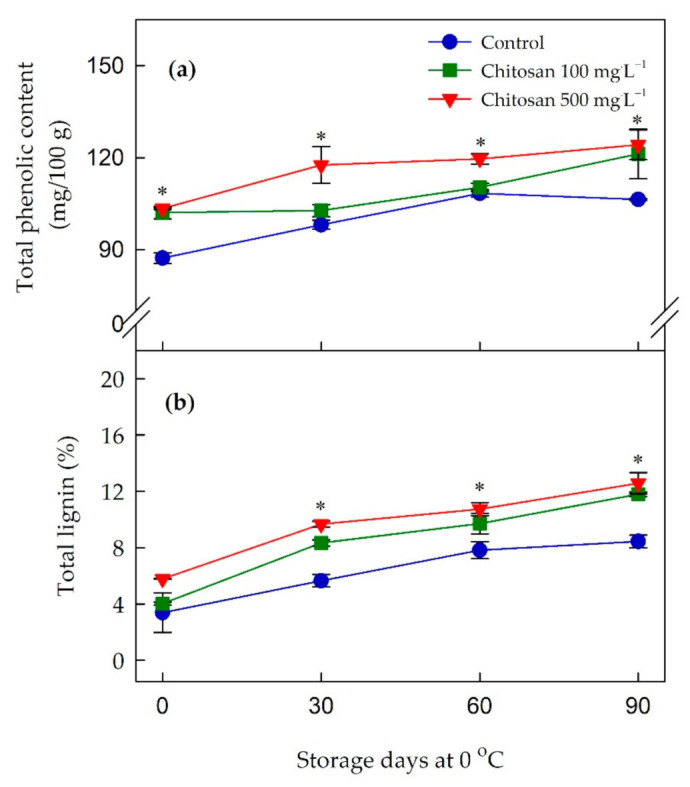
Effects of preharvest chitosan application on (**a**) total phenolic content and (**b**) total lignin content during cold storage in ‘Garmrok’ kiwifruit. Vertical bars indicate SE with n = 3. * indicates significant differences between treatments at each sampling date, according to the least significant difference (LSD) test at *p* ≤ 0.05.

In general, there was an increasing tendency in the content of total phenolic as the storage period prolonged in all the treatments (Figure 2a and Table S5). The application of preharvest chitosan at both concentrations caused the greatest content of total phenolic at harvest and this trend continued during the cold storage. Although 100 mg·L^−1^ chitosan-treated fruit maintained higher total phenolic content during the cold storage it was not statistically significant with untreated fruit at 30 and 60 days of storage. However, at the end of the storage period, the untreated fruit had the lowest total phenolic content (106.4 mg/100 g), while chitosan-treated fruit had total phenolic content of 121.3 and 124.2 mg/100 g for 100 and 500 mg·L^−1^ applications, respectively.

As shown in Figure 2b, the total lignin content increased over the whole storage in all the treatments (Table S5). There were no differences in total lignin content at harvest; however, the changes (*p* ≤ 0.05) in the content of total lignin between treatments became apparent from 30 days of storage. The application of preharvest chitosan at 500 mg·L^−1^ was associated with the greatest (*p* ≤ 0.05) total lignin content throughout the storage time, and this was followed by the 100 mg·L^−1^ chitosan-treated fruit.

### 3.6. Changes in Firmness

**Figure 3:**
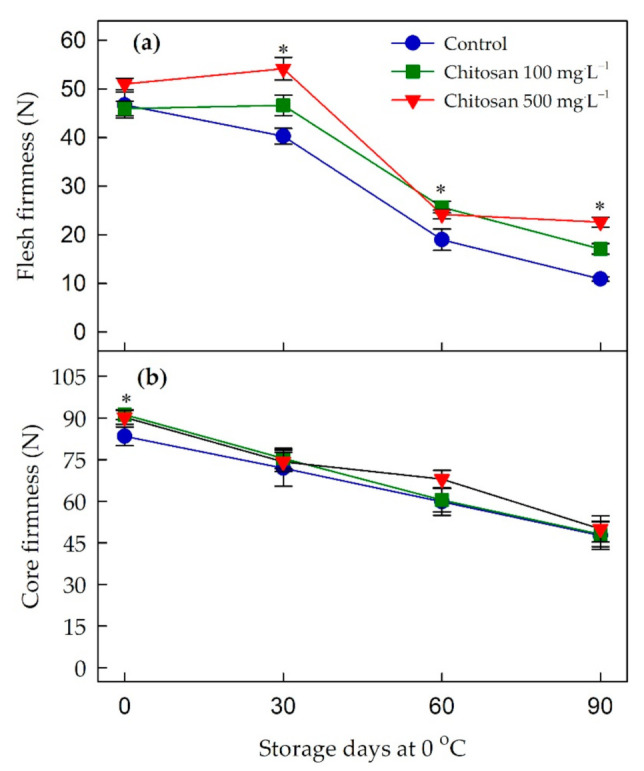
Effects of preharvest chitosan application on (**a**) flesh firmness and (**b**) core firmness during cold storage in ‘Garmrok’ kiwifruit. Vertical bars indicate SE with n = 10. * indicates significant differences between treatments at each sampling date, according to the least significant difference (LSD) test at *p* ≤ 0.05.

It is clear from Figure 3 and Table S3 that the firmness of kiwifruit (flesh and core) decreased gradually during the cold storage. At harvest, the firmness of flesh (Figure 3a) was not different between treatments, while the firmness of core (Figure 3b) was greatest (*p* ≤ 0.05) in the fruits treated with the preharvest chitosan. Throughout the whole storage, it was apparent that the treatment of chitosan at 100 and 500 mg·L^−1^ retained the higher flesh firmness when compared to that of control. Although the mean core firmness in preharvest chitosan-treated kiwifruits were higher than the control, they were not significantly different from the control fruit during the storage.

### 3.7. Expression of Ethylene Biosynthesis-, Cell Wall Modification- and Lignin Metabolism-Related Genes

**Figure 4:**
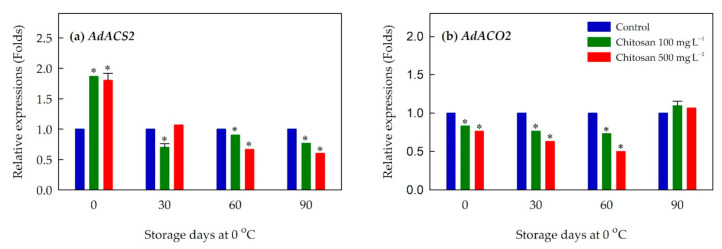
Effects of preharvest chitosan application on relative expression of ethylene biosynthesis-related genes (**a**) *AdACS2* and (**b**) *AdACO2* during cold storage in ‘Garmrok’ kiwifruit. Vertical bars indicate SE with n = 3. Actin gene was used as internal control and the quantitative RT-PCR data were analyzed using the 2^−ΔΔCT^ method. * Statistical difference caused by chitosan treatment was compared to untreated fruit (control) of each sampling date (*p* ≤ 0.05).

Expressions of several genes associated with ethylene biosynthesis were examined (Figure 4 and Table S6). In general, expression of *AdACS2* for the kiwifruit treated with preharvest chitosan at both concentrations was maintained at a relatively lower level than that of control during the storage (Figure 4a). This was apparent especially for the later stages of storage (i.e., 60 and 90 days). Another gene responsible for the biosynthesis of ethylene, *AdACO2*, displayed a similar pattern during the cold storage (Figure 4b). Preharvest treatment of chitosan at both concentrations suppressed expression of *AdACO2*, and this pattern of lower expression was observed until 60 days of cold storage.

**Figure 5:**
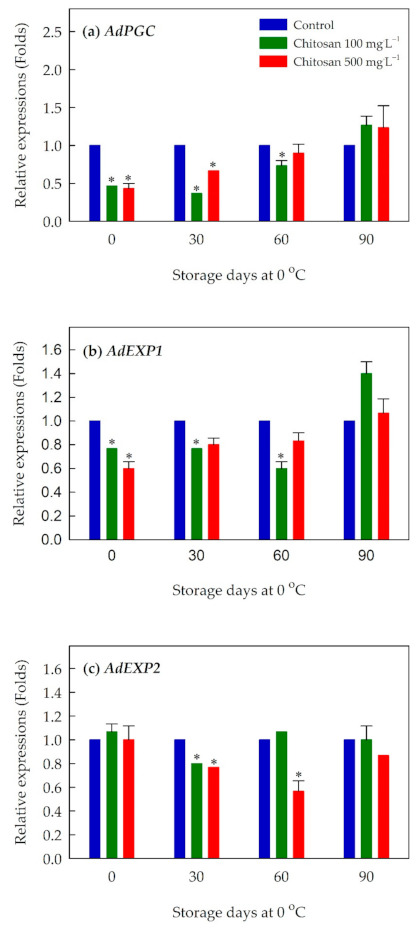
Effects of preharvest chitosan application on relative expression of cell wall-modification genes (**a**) *AdPGC*, (**b**) *AdEXP1*, and (**c**) *AdEXP2* during cold storage in ‘Garmrok’ kiwifruit. Vertical bars indicate SE with n = 3. Actin gene was used as internal control and the quantitative RT-PCR data were analyzed using the 2^−ΔΔCT^ method. * Statistical difference caused by chitosan treatment was compared to untreated fruit (control) of each sampling date (*p* ≤ 0.05).

We examined several genes related to cell wall modification and the pattern of expression is shown in Figure 5 and Table S6. The preharvest treatment of chitosan suppressed the expression of *AdPGC* (Figure 5a) and *AdEXP1* (Figure 5b) at 30 and 60 days of the cold storage. The suppressed expression of these two genes was observed primarily with lower concentration of chitosan (100 mg·L^−1^). The effect of suppressed expression for *AdEXP2* (Figure 5c) was greater with a higher concentration of chitosan (500 mg·L^−1^).

**Figure 6:**
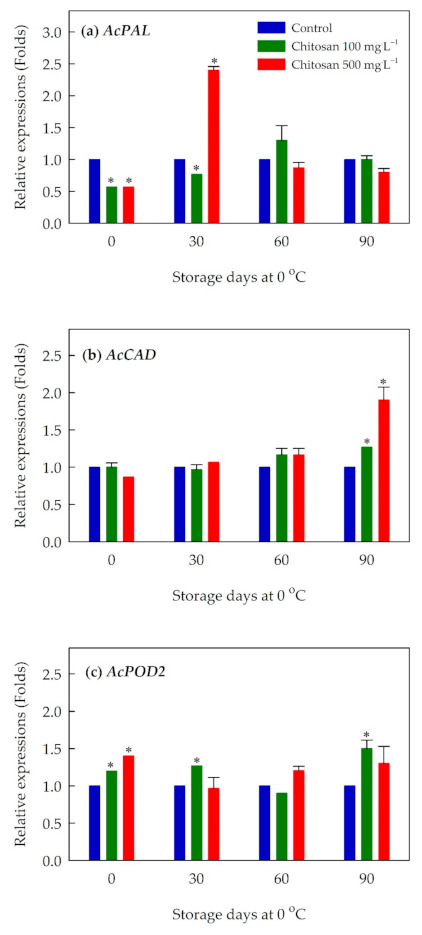
Effects of preharvest chitosan application on relative expression of lignin metabolism-related genes (**a**) *AcPAL*, (**b**) *AcCAD*, and (**c**) *AcPOD2* during cold storage in ‘Garmrok’ kiwifruit. Vertical bars indicate SE with n = 3. Actin gene was used as internal control and the quantitative RT-PCR data were analyzed using the 2^−ΔΔCT^ method. * Statistical difference caused by chitosan-treatment was compared to untreated fruit (control) of each sampling date (*p* ≤ 0.05).

As for genes associated with lignin metabolism, expression of *AcPAL*, a gene that is known to catalyze the first committed step in general phynylropanoid metabolism [32], was upregulated by the preharvest treatment of chitosan at both concentrations (Figure 6a and Table S6). The upregulation of *AcPAL* was most obvious at 30 days of cold storage. In addition, a gene considered to be an indicator of lignin biosynthesis [32], *AcCAD*, was upregulated at the end of cold storage (i.e., 90 days of storage) by both concentrations (Figure 6b and Table S6). A gene that is involved in the polymerization of monolignols to yield the lignin polymer [33], *AcPOD2*, was upregulated by the preharvest treatment of chitosan at both concentrations, especially toward the end of cold storage (Figure 6c and Table S6).

## 4. Discussion

### 4.1. Preharvest Application of Chitosan Increased the Weight of ‘Garmrok’ Kiwifruit at Harvest

In our previous study, we showed that the preharvest treatment of calcium chitosan (100 mg·L^−1^) increased the fruit weight with no effect on the size of kiwifruit at harvest [8]. A recent similar study demonstrated that the preharvest spray of chitosan increased the single fruit weight with the equivalent yield of kiwifruit without affecting the size of the fruit [22]. Here, we show that the application of preharvest chitosan also increased the fruit weight and delayed maturation and ripening of the kiwifruit. The influence of preharvest treatment of chitosan manifested throughout the cold storage is reflected on the physicochemical and molecular attributes in kiwifruit.

**Figure 7:**
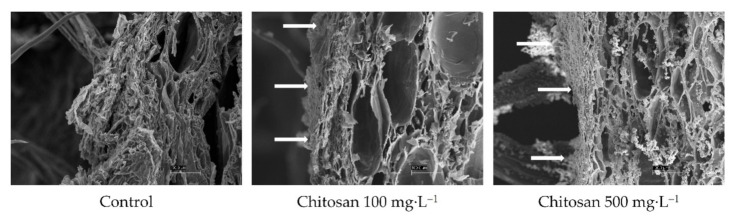
Scanning electron micrographs (SEMs) of preharvest chitosan-treated and untreated ‘Garmrok’ kiwifruit peel surfaces. The arrows indicate the chitosan film on kiwifruit peel after coating. All micrographs are at 500X and the scale bars in each image represent the lengths of 100 µm.

It was considered that the increased fruit weight by the treatment of chitosan is associated with the formation of a semipermeable barrier on the fruit surface that may have reduced the water loss from transpiration and respiration [8]. When we examined the surface of the ‘Garmrok’ kiwifruit peel under a scanning electron microscope, this semipermeable barrier-forming characteristic of chitosan was observed as a coated film on the kiwifruit peel (Figure 7). The film coating of chitosan on the fruit surface can absorbs moisture due to the hydrophilic nature of chitosan solution [34] and this could be attributed to the increased weight of kiwifruit, which in turn reduced water loss from fresh fruit. Alternatively, the increased weight of chitosan-treated kiwifruit may be attributed to the activity as a biostimulant and growth elicitor, as demonstrated in *Capsicum annuum* ‘Yolo Wonder’ bell pepper [35], *Solanum lycopersicon* ‘Faridah’ tomato [36], and *A. deliciosa* ‘Guichang’ kiwifruit [22]. Future investigations on the detailed mechanistic insights as to the increased weight of fruits at harvest pretreated by chitosan further awaits.

### 4.2. Preharvest Application of Chitosan Is Associated with the Reduced Production of Ethylene and Rate of Respiration during Cold Storage

During the cold storage, we observed a noticeable effect of preharvest chitosan application on the reduction of ethylene production and respiration rate of ‘Garmrok’ kiwifruit. The reduced ethylene production and respiration rate can be explained by the film-forming property of chitosan [17,19]. Chitosan has excellent selective permeability to the respiratory gases, particularly by blocking the oxygen that reduces respiration [20]. Kiwifruit is a climacteric fruit and the ripening process is largely regulated by the plant hormone ethylene. Ethylene production in climacteric fruit is mainly governed by the activity of two enzymes, i.e., 1-aminocyclopropane-1-carboxylic acid (ACC) synthase (ACS) and ACC oxidase (ACO) [37,38]. The conversion of ACC to ethylene by ACO is O_2_-dependent [37,38]. The enzyme activity of ACO is suppressed by low O_2_ and high CO_2_, while high CO_2_ concentrations prevent the auto-induction of ACS [37,38,39]. The effect of low O_2_ and high CO_2_ concentrations during storage on ethylene production and ripening for the shelf-life has been well reported in *A. deliciosa* ‘Hayward’ kiwifruit [39,40] and also in *Pyrus communis* ‘Conference’ pear [41].

The application of preharvest chitosan that acted as a barrier of oxygen might have affected the low production of ethylene in the kiwifruit by suppressing the key genes in the ethylene biosynthesis. Recently, He et al. [42] also observed that chitosan oligosaccharides (COS) suppressed the expression of genes involved in the ethylene biosynthesis (*FaACS* and *FaACO*), which reduced the softness and increased the shelf-life of strawberry fruit (*Fragaria ananassa* ‘Qingxiang’). In addition, the resultant reduced production of ethylene and respiration rates might have positively influenced the retention of various quality attributes that prolonged the postharvest life of ‘Garmrok’ kiwifruit.

### 4.3. Preharvest Application of Chitosan Reduced the Weight Loss of ‘Garmrok’ Kiwifruit during Cold Storage

The proper status of water in fresh fruit before and during postharvest handling plays a critical role in maintaining the quality of the crop [36]. In general, weight loss in fresh fruits is caused by water loss associated with the metabolic activities occurring during respiration and transpiration processes [19]. In this study, we show that the application of preharvest chitosan reduces the weight loss of ‘Garmrok’ kiwifruit during cold storage. Previous studies have demonstrated that several features of chitosan treatment are linked to the reduction of weight loss in various fruits [13,17,19,36,43,44,45,46]. These include chitosan coatings not just as a barrier of transpiration, but also as a protectant and a wound sealer of fruit skin from mechanical injuries that delayed dehydration.

### 4.4. Preharvest Application of Chitosan Possesses Maturity- and Ripening-Delaying Effects on ‘Garmrok’ Kiwifruit

Soluble solids content (SSC), titratable acidity (TA), and total sugar and acid contents are important chemical attributes associated with the edible quality of ripe kiwifruit and are used as maturity- and ripening-related indices in quality measurements [5]. The increase in SSC during fruit ripening could be attributed to the breakdown of carbohydrates into simple sugars [5,19,44]. The reduction in TA during fruit ripening, on the other hand, indicates the degradation of organic acids, which are used as respiratory substrates [5,17,44]. It has been shown that low respiration and ethylene production repress the hydrolysis of carbohydrates, which results in a low SSC, total sugar contents, and delayed ripening [36]. In another report, the ratio of fructose + glucose/sucrose at harvest, a possible indicator of invertase activity [31], was shown to be associated with maturity- and ripening-delaying effects by preharvest application of chitosan, which supports our current results and also our previous study [8].

Our results revealed the slower increasing rate of SSC with lower values of SSC at the end of cold storage in ‘Garmrok’ kiwifruit when treated with preharvest chitosan. In addition, the kiwifruit treated with preharvest chitosan displayed a slower increase in total sugar content followed by the lowest total sugar content at the end of cold storage. These changes may have to do with the reduced rates of respiration and ethylene production, which in turn slowed the metabolic activity in preharvest chitosan-treated ‘Garmrok’ kiwifruit. Furthermore, the slow degradation of organic acids observed in our study may have resulted from the reduced respiration rate, which slowed the reduction in TA and total acid content. Our findings are consistent with the results of those observed in other fruits by preharvest treatment with chitosan [17,19,44,45,46].

### 4.5. Preharvest Application of Chitosan Might Improve the Antioxidant Activity of ‘Garmrok’ Kiwifruit by Positively Affecting Phenolic Metabolism

Phenolic compounds, the major secondary metabolites in plants, are responsible for the flavor and color of fruits with antioxidant potential [22,26,36,42]. The (+)-catechin, chlorogenic acid, rutin, (−)-epicatechin, quercetin, and tannin are the major phenolics identified in kiwifruit [26] and are responsible for the antioxidant capacity of fruit while being involved in the antioxidant metabolism [22,26]. Over the 90 days of storage period at 0 °C, preharvest application of chitosan increased the total phenolic content in ‘Garmrok’ kiwifruit, as similarly shown in other fruits treated with preharvest chitosan [22,34,36,42,45,47,48]. The low level of total phenolic content observed in the untreated kiwifruit might be due to the rapid breakdown of cell structure compared with the chitosan-treated kiwifruits. The fact that kiwifruit treated with preharvest chitosan that retained higher total content of phenolic compounds may be associated with the reduced activity of polyphenol oxidase (PPO) that oxidizes the phenolic compounds. The increased content of total phenolic compounds could possibly have obtained the antioxidant capacity of kiwifruit during the cold storage [36,42], which not only elicited immune responses but also improved the quality of ‘Garmrok’ kiwifruit during cold storage that delayed the ripening.

### 4.6. Preharvest Application of Chitosan Maintained the Firmness of ‘Garmrok’ Kiwifruit during Cold Storage

Loss of fruit firmness is a deleterious quality attribute that determines the postharvest life, fruit quality, commercial value, and consumer acceptability. In our study, preharvest application of chitosan exerted a beneficial effect on kiwifruit firmness during the cold storage. These results are in agreement with those obtained in our previous study on *A. deliciosa* ‘Garmrok’ kiwifruit [8] and Zhang et al. [22] on *A. deliciosa* ‘Guichang’ kiwifruit. It was considered that maintenance of firmness by chitosan coatings results from the formation of a semipermeable film on fruit surface that can act as a protective barrier to reduce respiration rate, thereby slowing the metabolic activity and textural changes [17,19,22,44,46,49]. As kiwifruit is a typical climacteric fruit, the reduced production of respiration and ethylene affected by chitosan treatment appear to be linked to the kiwifruit softening. Whether ethylene directly regulates expression of cell wall-modifying enzymes in kiwifruit, as observed in other climacteric, fruit remains to be investigated.

In addition, fruit firmness is closely associated with changes in the biochemical properties of the cell wall. Polygalacturonase (PG) is a key enzyme involved in cell wall modifications during fruit ripening, with its important functions in degradation of soluble pectin and depolymerization of solubilized pectin [50,51,52]. In kiwifruit, there are three polygalacturonase genes, namely, *PGA*, *PGB*, and *PGC*. Of these, *PGC* is a predominant gene expressed throughout the softening of kiwifruit [50,51,53]. The suppression of the *AdPGC* gene in preharvest chitosan-treated ‘Garmrok’ kiwifruit during the early phase of storage (i.e., 30 and 60 days) may be associated with the delayed kiwifruit softening and retained firmness. Expansins (EXPs) are non-enzymatic proteins that have been considered to play a role in loosening and extension of the cell wall (the cellulose-xyloglucan hydrogen bonds) [50,52,54]. Yang et al. [54] reported that two expansin genes *AdEXP1* and *AdEXP2* are actively involved in kiwifruit (*A. deliciosa* ‘Bruno’) ripening and softening. In our study, we observed a negative effect on expression of expansin genes (*AdEXP1* and *AdEXP2*) during the postharvest storage of ‘Garmrok’ kiwifruit. Thus, preharvest chitosan treatment was effective in delaying fruit softening, maintaining kiwifruit firmness, and extend their storage life by possibly inhibiting the activities of EXPs.

Further, a higher flesh firmness of ‘Garmrok’ kiwifruit treated with preharvest chitosan can be ascribed to the increase in lignin content. Lignin is one of the most abundant polyphenolic polymers in higher plants that mostly functions as a structural material which enhances the strength and rigidity of plant cells [12,55]. The increase of total lignin content in the chitosan-treated ‘Garmrok’ kiwifruit during the cold storage indicates that preharvest chitosan could induce lignin biosynthesis, as shown by He et al. [42] and Saavedra et al. [48]. Lignin biosynthesis involves the activities of three major enzymes (PAL, CAD, and POD), as well as the coordinated expression of these enzyme genes [12,32,33,55,56]. In our study, the higher expression of *AcPAL* during the early period of storage may have influenced the lignin accumulation in the preharvest chitosan-treated kiwifruit. In addition, the increased level of expression in *AcCAD* and *AcPOD2* at the later stage of storage is associated with the increased content of lignin in the preharvest chitosan-treated kiwifruit. Collectively, expression of lignin metabolism-related genes affected by preharvest chitosan treatment may have enhanced lignin metabolism that conferred greater firmness in ‘Garmrok’ kiwifruits.

## 5. Conclusions

Our results demonstrate that *A. deliciosa* ‘Garmrok’ kiwifruit can be stored at 0 °C for more than two months when preharvest chitosan was applied to kiwifruit. Preharvest application of chitosan reduced weight loss, maintained firmness by modulating the expression of ethylene biosynthesis, cell wall modification, and lignin metabolism-related genes, possibly evoked antioxidant activity by phenolic metabolism, and displayed a maturity- and the ripening-delaying effect that improved the overall quality and extended the storage life of kiwifruit. This work also revealed that the treatment with 500 mg·L^−1^ chitosan was more effective in maintaining overall physicochemical attributes of ‘Garmrok’ kiwifruit during postharvest storage. The results open a promising strategy for maintaining the postharvest properties of kiwifruit. Detailed mechanistic insights that underlie physicochemical attributes need to be further investigated. In addition, the possible effect on sensory attributes of chitosan-coated kiwifruit and lignin accumulation for the consumer’s acceptance should be taken into consideration in future studies.

## Figures and Tables

**Table 1:** Effects of preharvest chitosan application on fruit weight and fruit size at harvest in ‘Garmrok’ kiwifruit. Experimental data represent means ± standard error with n = 10. * in each column indicate significant differences between treatments on harvest day, according to the least significant difference (LSD) test at *p* ≤ 0.05.

Treatment	Fruit Weight (g)	Fruit Size (mm)		
		Length	Longitudinal Width	Transverse Width
Control	81.49 ± 1.1	55.17 ± 0.7	55.85 ± 0.7	52.06 ± 0.6 *
Chitosan 100 mg·L^−1^	86.27 ± 2.4 *	54.72 ± 1.2	54.25 ± 0.5	50.28 ± 0.4
Chitosan 500 mg·L^−1^	91.79 ± 2.9 *	55.17 ± 1.1	55.45 ± 0.5	51.86 ± 0.6

**Table 2:** Effects of preharvest chitosan application on fruit weight loss in ‘Garmrok’ kiwifruit during cold storage. Experimental data represent means ± standard error with n = 10. * in each column indicate significant differences between treatments at each sampling date, according to the least significant difference (LSD) test at *p* ≤ 0.05.

Treatment	Storage Days at 0 °C			
	0	30	60	90
	Fruit weight loss (%)			
Control	0.0±0.0	0.9 ± 0.0	1.5 ± 0.1	2.3 ± 0.1
Chitosan 100 mg·L^−1^	0.0±0.0	0.5 ± 0.1 *	1.0 ± 0.1 *	1.8 ± 0.1 *
Chitosan 500 mg·L^−1^	0.0±0.0	0.6 ± 0.1 *	1.2 ± 0.1 *	2.2 ± 0.2

**Table 3:** Effects of preharvest chitosan application on soluble solids content, titratable acidity, total sugar content (HPLC analysis), total acid content (HPLC analysis), and their ratios in ‘Garmrok’ kiwifruit during cold storage. Experimental data represent means ± standard error with n = 10 (SSC) and n = 3 (titratable acidity, total sugar content, total acid content, and their ratios). *, in each column indicate significant differences between treatments at each sampling date, according to the least significant difference (LSD) test at *p* ≤ 0.05.

Treatment	Storage Days at 0 °C			
	0	30	60	90
	Soluble solids content (SSC, %)			
Control	6.2 ± 0.2	9.6 ± 0.1	11.8 ± 0.2	12.7 ± 0.1
Chitosan 100 mg·L^−1^	6.0 ± 0.2	9.3 ± 0.2 *	11.7 ± 0.2	11.9 ± 0.1 *
Chitosan 500 mg·L^−1^	6.0 ± 0.2	9.9 ± 0.2	11.4 ± 0.1	11.6 ± 0.3 *
	Titratable acidity (TA, %)			
Control	1.7 ± 0.0	1.5 ± 0.0	1.2 ± 0.1	1.1 ± 0.0
Chitosan 100 mg·L^−1^	1.9 ± 0.1 *	1.5 ± 0.0	1.3 ± 0.0 *	1.0 ± 0.0
Chitosan 500 mg·L^−1^	1.8 ± 0.0 *	1.5 ± 0.0	1.2 ± 0.0	0.9 ± 0.0 *
	Total sugar content (g/100 g)			
Control	6.9 ± 0.2	9.7 ± 0.4	9.2 ± 0.5	9.4 ± 0.5
Chitosan 100 mg·L^−1^	6.8 ± 0.2	9.2 ± 0.3	8.9 ± 0.2	9.2 ± 0.3
Chitosan 500 mg·L^−1^	6.7 ± 0.2	8.4 ± 0.2 *	8.5 ± 0.4	8.1 ± 0.2 *
	Total acid content (g/100 g)			
Control	1.3 ± 0.0	1.3 ± 0.0	1.2 ± 0.0	1.3 ± 0.0
Chitosan 100 mg·L^−1^	1.4 ± 0.0*	1.4 ± 0.0 *	1.3 ± 0.0 *	1.4 ± 0.0 *
Chitosan 500 mg·L^−1^	1.4 ± 0.0*	1.3 ± 0.0	1.3 ± 0.0 *	1.3 ± 0.0
	Fructose + glucose/sucrose ratio			
Control	4.3 ± 0.3	2.3 ± 0.2	2.5 ± 0.0	2.6 ± 0.1
Chitosan 100 mg·L^−1^	3.6 ± 0.2*	2.1 ± 0.1	2.3 ± 0.3	3.1 ± 0.7
Chitosan 500 mg·L^−1^	3.4 ± 0.1*	2.1 ± 0.2	2.4 ± 0.0	2.6 ± 0.2
	Citric acid/quinic acid ratio			
Control	1.2 ± 0.0	1.2 ± 0.0	1.2 ± 0.0	1.2 ± 0.0
Chitosan 100 mg·L^−1^	1.3 ± 0.0 *	1.2 ± 0.0	1.1 ± 0.0 *	1.1 ± 0.0
Chitosan 500 mg·L^−1^	1.4 ± 0.0 *	1.1 ± 0.1	1.2 ± 0.0	0.9 ± 0.1

## Supplementary Materials

The following are available online at https://www.mdpi.com/2304-8158/10/2/373/s1, Table S1: Effects of preharvest chitosan application on sugars (fructose, glucose, and sucrose) and organic acids (oxalic, quinic, malic, and citric) (HPLC analysis) in ‘Garmrok’ kiwifruit during cold storage. Experimental data represent means ± standard error with n = 3. *, in each column indicate significant differences between treatments at each sampling date, according to the least significant difference (LSD) test at *p* ≤ 0.05, Table S2: The primer sequences and gene information used in this study, Table S3: Ethylene production, respiration, fruit weight loss, flesh firmness and core firmness of ‘Garmrok’ kiwifruit as affected by chitosan-treatment and days of storage at 0 °C, Table S4: Soluble solids content (SSC), titratable acidity (TA), total sugar content (HPLC analysis), total acid content (HPLC analysis), and their ratios of ‘Garmrok’ kiwifruit as affected by chitosan-treatment and days of storage at 0 °C, Table S5: Total phenolic content and total lignin content of ‘Garmrok’ kiwifruit as affected by chitosan-treatment and days of storage at 0 °C, Table S6: Relative expression of ethylene biosynthesis-related genes (*AdACS2* and *AdACO2*), cell wall-modification genes (*AdPGC*, *AdEXP1*, and *AdEXP2*), and lignin metabolism-related genes (*AcPAL*, *AcCAD*, and *AcPOD2*) of ‘Garmrok’ kiwifruit as affected by chitosan-treatment and days of storage at 0 °C.
