# Supplementary material for: Preharvest Application of Chitosan Improves the Postharvest Life of ‘Garmrok’ Kiwifruit through the Modulation of Genes Related to Ethylene Biosynthesis, Cell Wall Modification and Lignin Metabolism
Source: Foods. 2021 Feb 9;10(2):373. doi: 10.3390/foods10020373 (PMC7915756; doi:10.3390/foods10020373)
Supplement: Supplementary file 1 [file foods-10-00373-s001.pdf]

foods

Article

Preharvest Application of Chitosan Improves the Postharvest Life of 'Garmrok' Kiwifruit through the Modulation of Genes Related to Ethylene Biosynthesis, Cell Wall Modification, and Lignin Metabolism

H. M. Prathibhani C. Kumarihami [1], Jin Gook Kim [1,2], Yun-Hee Kim [3], Mockhee Lee [4], Young-Suk Lee [5], Yong-Bum Kwack [6] and Joonyup Kim [7,*]

[1] Division of Applied Life Science, Graduate School, Gyeongsang National University, Jinju 52828, Korea; prathibhanick@gmail.com
[2] Institute of Agriculture and Life Science, Gyeongsang National University, Jinju 52828, Korea; jgkim119@gnu.ac.kr
[3] Department of Biology Education, Gyeongsang National University, Jinju 52828, Korea; cefle@gnu.ac.kr
[4] Namhae Branch, National Institute of Horticultural and Herbal Science, Rural Development Administration, Namhae 52430, Korea; mockey92@korea.kr
[5] Department of Horticulture Research, Gyeongsangnam-do Agricultural Research and Extension Service, Jinju 52733, Korea; yseve77@korea.kr
[6] Department of Fruit Science, Korea National College of Agriculture and Fisheries, Jeonju 54874, Korea; kwack@korea.kr
[7] Department of Horticultural Science, Chungnam National University, Daejeon 34134, Korea; jykim12@cnu.ac.kr
* Correspondence: jykim12@cnu.ac.kr; Tel.: +82-42-821-5738

Table S1. Effects of preharvest chitosan application on sugars (fructose, glucose, and sucrose) and organic acids (oxalic, quinic, malic, and citric) (HPLC analysis) in 'Garmrok' kiwifruit during cold storage. Experimental data represent means ± standard error with n = 3. *, in each column indicate significant differences between treatments at each sampling date, according to the least significant difference (LSD) test at $p \leq 0.05$.

**Treatment**	**Storage days at 0 °C**			
	**0**	**30**	**60**	**90**
	Fructose (g/100 g)			
Control	2.7±0.2	3.4±0.2	3.1±0.2	3.3±0.2
Chitosan 100 mg·L^{-1}	2.3±0.1	3.1±0.1	3.0±0.1	3.3±0.4
Chitosan 500 mg·L^{-1}	2.7±0.1	2.7±0.2*	2.6±0.1*	2.9±0.1
	Glucose (g/100 g)			
Control	2.8±0.	3.2±0.1	3.5±0.2	3.5±0.3
Chitosan 100 mg·L^{-1}	2.9±0.1	3.1±0.1	3.2±0.3	3.4±0.1
Chitosan 500 mg·L^{-1}	2.4±0.2*	2.9±0.2	3.4±0.3	3.0±0.1*
	Sucrose (g/100 g)			
Control	1.3±0.0	3.0±0.2	2.6±0.1	2.6±0.1
Chitosan 100 mg·L^{-1}	1.5±0.1	3.0±0.2	2.7±0.2	2.5±0.6
Chitosan 500 mg·L^{-1}	1.5±0.0	2.7±0.1	2.5±0.1	2.3±0.2
	Oxalic acid (g/100 g)			
Control	0.02±0.0	0.01±0.0	0.01±0.0	0.01±0.0
Chitosan 100 mg·L^{-1}	0.01±0.0*	0.01±0.0	0.02±0.0*	0.01±0.0
Chitosan 500 mg·L^{-1}	0.01±0.0*	0.02±0.0	0.01±0.0	0.01±0.0
	Quinic acid (g/100 g)			
Control	0.6±0.0	0.5±0.0	0.5±0.0	0.5±0.0
Chitosan 100 mg·L^{-1}	0.5±0.0	0.6±0.0	0.5±0.0	0.6±0.0*
Chitosan 500 mg·L^{-1}	0.6±0.0	0.6±0.0	0.5±0.0	0.5±0.0
	Malic acid (g/100 g)			
Control	0.1±0.0	0.1±0.0	0.1±0.0	0.1±0.0
Chitosan 100 mg·L^{-1}	0.1±0.0	0.1±0.0	0.2±0.0*	0.2±0.0
Chitosan 500 mg·L^{-1}	0.1±0.0	0.1±0.0	0.1±0.0	0.1±0.0
	Citric acid (g/100 g)			
Control	0.6±0.0	0.6±0.0	0.6±0.0	0.6±0.0
Chitosan 100 mg·L^{-1}	0.7±0.0*	0.7±0.0*	0.6±0.0	0.6±0.0
Chitosan 500 mg·L^{-1}	0.8±0.0*	0.6±0.0	0.7±0.0*	0.5±0.1

Table S2. The primer sequences and gene information used in this study.

Gene name	Primer sequence (5′-3′)		Gene ID	Annotation
AdACS2	Forward	TCCACCTCATCAG-TGACGAA	KY607911.1	*A. deliciosa* ACC synthase 2
	Reverse	ACCACATCGTCGTT-GGAGTA		
AdACO2	Forward	ATGAAGGCCATGGAAAGTGC	HQ293205.1	*A. deliciosa* ACC oxidase 2
	Reverse	TGTGAACTCCAA-GACCACCA		
AdPGC	Forward	GGCAG-TGCTTCTGGTTTAGT	AF152756.1	*A. deliciosa* polygalac-turonase C
	Reverse	CACCACAGGCTCAA-GCAAAT		
AdEXP1	Forward	GAT-TTCAGGTTTCCCGCGTT	AY390358.2	*A. deliciosa* expansin 1
	Reverse	ACATGCCTCTTTTCTGCAGC		
AdEXP2	Forward	AAGTTACAATGTGGCGCCTG	DQ915940.1	*A. deliciosa* expansin 2
	Reverse	ACCTCTGCTTTA-GAAGGCCA		
AcPAL	Forward	TCGGAGCTT-GTCAACGACT	Achn066341	*A. chinensis* phenylalanine ammonia-lyase
	Reverse	GTTTTTCTTGATGA-GATGAGGC		
AcCAD	Forward	GCGTTTTTCATACAG-CATCTC	Achn241151	*A. chinensis* cinnam-yl-alcohol dehydrogenase
	Reverse	TTCATCAACTACCAC-TTCAGGA		
AcPOD2	Forward	AAGAT-TCATGCCCACAAGC	Achn150921	*A. chinensis* peroxidase 2
	Reverse	AGGGCAC-TCTCTCTCTACAGC		
AdACT1	Forward	TGGGTACATGAATT-GGAGGGA	EF063572.1	*A. deliciosa* actin 1
	Reverse	TCCAA-GAAAAGGGACCCTT-GA		

Table S3. Ethylene production, respiration, fruit weight loss, flesh firmness and core firmness of 'Garmrok' kiwifruit as affected by chitosan-treatment and days of storage at 0 °C.

Treatment (A)	Storage days at 0 °C (B)	C_2H_4 (μl·kg^{-1}·h^{-1})	CO_2 (mg·kg^{-1}·h^{-1})	Fruit weight loss (%)	Flesh firmness (N)	Core firmness (N)
Control	0	7.11±0.2	15.88±0.7	0.0±0.0	46.7±2.7	83.4±3.4
	30	6.99±0.3	21.9±1.5	0.9±0.0	40.3±1.6	71.9±6.5
	60	6.72±0.3	22.4±2.4	1.5±0.1	19.0±2.2	59.8±4.9
	90	8.54±0.1	20.0±1.5	2.3±0.1	10.9±0.4	47.7±5.1
Chitosan 100 mg·L^{-1}	0	7.07±0.6	15.45±0.4	0.0±0.0	46.0±1.5	91.2±1.8
	30	6.28±0.4	17.8±0.6	0.5±0.1	46.6±2.1	75.4±3.7
	60	5.92±0.3	23.5±1.9	1.0±0.1	25.7±1.2	60.4±4.2
	90	6.95±0.1	16.4±1.6	1.8±0.1	17.1±1.1	48.1±4.5
Chitosan 500 mg·L^{-1}	0	7.41±0.1	15.41±0.6	0.0±0.0	51.0±1.2	90.2±2.5
	30	7.76±0.0	16.9±1.1	0.6±0.1	54.2±2.3	74.2±3.5
	60	5.78±0.3	22.5±1.3	1.2±0.1	24.2±0.9	68.0±3.1
	90	7.02±0.3	14.3±0.8	2.2±0.2	22.6±1.0	50.0±4.7
F-test	A	** y	*	***	***	NS
	B	***	***	***	***	***
	A x B	**	NS	NS	**	NS

[y]NS,*,**,***Nonsignificant or significant at $p \leq 0.05$, 0.01 or 0.001 respectively.

Table S4. Soluble solids content (SSC), titratable acidity (TA), total sugar content (HPLC analysis), total acid content (HPLC analysis), and their ratios of 'Garmrok' kiwifruit as affected by chitosan-treatment and days of storage at 0 °C.

Treatment (A)	Storage days at 0 °C (B)	SSC (%)	TA (%)	Total sugar content (g/100 g)	Total acid content (g/100 g)	Fructose + glucose/sucrose ratio	Citric acid/quinic acid ratio
Control	0	6.2±0.2	1.7±0.0	6.9±0.2	1.3±0.0	4.3±0.3	1.2±0.0
	30	9.6±0.1	1.5±0.0	9.7±0.4	1.3±0.0	2.3±0.2	1.2±0.0
	60	11.8±0.2	1.2±0.1	9.2±0.5	1.2±0.0	2.5±0.0	1.2±0.0
	90	12.7±0.1	1.1±0.0	9.4±0.5	1.3±0.0	2.6±0.1	1.2±0.0
Chitosan 100 mg·L^{-1}	0	6.0±0.2	1.9±0.1	6.8±0.2	1.4±0.0	3.6±0.2	1.3±0.0
	30	9.3±0.2	1.5±0.0	9.2±0.3	1.4±0.0	2.1±0.1	1.2±0.0
	60	11.7±0.2	1.3±0.0	8.9±0.2	1.3±0.0	2.3±0.3	1.1±0.0
	90	11.9±0.1	1.0±0.0	9.2±0.3	1.4±0.0	3.1±0.7	1.1±0.0
Chitosan 500 mg·L^{-1}	0	6.0±0.2	1.8±0.0	6.7±0.2	1.4±0.0	3.4±0.1	1.4±0.0
	30	9.9±0.2	1.5±0.0	8.4±0.2	1.3±0.0	2.1±0.2	1.1±0.1
	60	11.4±0.1	1.2±0.0	8.5±0.4	1.3±0.0	2.4±0.0	1.2±0.0
	90	11.6±0.3	0.9±0.0	8.1±0.2	1.3±0.0	2.6±0.2	0.9±0.1
F-test	A	** y	*	***	***	NS	NS
	B	***	***	***	**	***	**
	A x B	**	**	NS	NS	NS	**

^{y}NS,*,**,***Nonsignificant or significant at $p \leq 0.05$, 0.01 or 0.001 respectively.

Table S5. Total phenolic content and total lignin content of 'Garmrok' kiwifruit as affected by chitosan-treatment and days of storage at 0 °C.

Treatment (A)	Storage days at 0 °C (B)	Total phenolic content (mg/100 g)	Total lignin content (%)
Control	0	87.3±1.7	3.4±1.4
	30	98.2±1.4	5.6±0.4
	60	108.4±1.2	7.8±0.6
	90	106.4±0.3	8.4±0.4
Chitosan 100 $mg \cdot L^{-1}$	0	102.1±2.0	4.0±0.1
	30	102.8±2.0	8.3±0.2
	60	110.4±1.3	9.7±0.7
	90	121.3±8.1	11.8±0.2
Chitosan 500 $mg \cdot L^{-1}$	0	103.4±0.3	5.8±0.0
	30	117.6±6.0	9.7±0.2
	60	119.6±1.7	10.7±0.5
	90	124.2±4.9	12.6±0.8
F-test	A	*** [y]	***
	B	***	***
	A x B	NS	NS

[y]NS,*,**,***Nonsignificant or significant at $p \leq 0.05$, 0.01 or 0.001 respectively.

Table S6. Relative expression of ethylene biosynthesis-related genes (*AdACS2* and *AdACO2*), cell wall-modification genes (*AdPGC*, *AdEXP1*, and *AdEXP2*), and lignin metabolism-related genes (*AcPAL*, *AcCAD*, and *AcPOD2*) of 'Garmrok' kiwifruit as affected by chitosan-treatment and days of storage at 0 °C.

Treatment (A)	Storage days at 0 °C (B)	*AdACS2*	*AdACO2*	*AdPGC*	*AdEXP1*	*AdEXP2*	*AcPAL*	*AcCAD*	*AcPOD2*
Control	0	1.0±0.0	1.0±0.0	1.0±0.0	1.0±0.0	1.0±0.0	1.0±0.0	1.0±0.0	1.0±0.0
	30	1.0±0.0	1.0±0.0	1.0±0.0	1.0±0.0	1.0±0.0	1.0±0.0	1.0±0.0	1.0±0.0
	60	1.0±0.0	1.0±0.0	1.0±0.0	1.0±0.0	1.0±0.0	1.0±0.0	1.0±0.0	1.0±0.0
	90	1.0±0.0	1.0±0.0	1.0±0.0	1.0±0.0	1.0±0.0	1.0±0.0	1.0±0.0	1.0±0.0
Chitosan 100 mg·L^{-1}	0	1.9±0.0	0.8±0.0	0.5±0.0	0.8±0.0	1.1±0.1	0.6±0.0	1.0±0.1	1.2±0.0
	30	0.7±0.1	0.8±0.0	0.4±0.0	0.8±0.0	0.8±0.0	0.8±0.0	1.0±0.1	1.3±0.0
	60	0.9±0.0	0.7±0.0	0.7±0.1	0.6±0.1	1.1±0.0	1.3±0.2	1.2±0.1	0.9±0.0
	90	0.8±0.0	1.1±0.1	1.3±0.1	1.4±0.1	1.0±0.1	1.0±0.1	1.3±0.0	1.5±0.1
F-test	A	** y	***	***	**	NS	NS	**	***
	B	***	***	***	***	*	**	*	***
	A x B	***	***	***	***	*	**	*	***
Control	0	1.0±0.0	1.0±0.0	1.0±0.0	1.0±0.0	1.0±0.0	1.0±0.0	1.0±0.0	1.0±0.0
	30	1.0±0.0	1.0±0.0	1.0±0.0	1.0±0.0	1.0±0.0	1.0±0.0	1.0±0.0	1.0±0.0
	60	1.0±0.0	1.0±0.0	1.0±0.0	1.0±0.0	1.0±0.0	1.0±0.0	1.0±0.0	1.0±0.0
	90	1.0±0.0	1.0±0.0	1.0±0.0	1.0±0.0	1.0±0.0	1.0±0.0	1.0±0.0	1.0±0.0
Chitosan 500 mg·L^{-1}	0	1.8±0.1	0.8±0.0	0.4±0.1	0.6±0.1	1.0±0.1	0.6±0.0	0.9±0.0	1.4±0.0
	30	1.1±0.0	0.6±0.0	0.7±0.0	0.8±0.1	0.8±0.0	2.4±0.1	1.1±0.0	1.0±0.1
	60	0.7±0.0	0.5±0.0	0.9±0.1	0.8±0.1	0.6±0.1	0.9±0.1	1.2±0.1	1.2±0.1
	90	0.6±0.0	1.1±0.0	1.2±0.3	1.1±0.1	0.9±0.0	0.8±0.1	1.9±0.2	1.3±0.2
F-test	A	NS	***	*	***	***	***	***	**
	B	***	***	*	**	**	***	***	NS
	A x B	***	***	*	**	**	***	***	NS

[y]NS,*,**,***Nonsignificant or significant at $p \leq 0.05$, 0.01 or 0.001 respectively.
